# Oral Cancer Malnutrition Impacts Weight and Quality of Life

**DOI:** 10.3390/nu7042145

**Published:** 2015-03-27

**Authors:** Nils-Claudius Gellrich, Jörg Handschel, Henrik Holtmann, Gertrud Krüskemper

**Affiliations:** 1 Department of Cranio-Maxillofacial Surgery, Hannover Medical School, Carl-Neuberg-Street 1, Hannover D-30625, Germany; E-Mail: gellrich.nils-claudius@mh-hannover.de; 2 Department for Oral and Maxillofacial Surgery, Heinrich Heine University of Düsseldorf, Moorenstr. 5, D-40225 Düsseldorf, Germany; E-Mail: Henrik.Holtmann@med.uni-duesseldorf.de; 3 Department of Medical Psychology, Ruhr University of Bochum, Universitätsstr. 150, Building MA 0/145, D-44780 Bochum, Germany; E-Mail: krueskem@uni-duesseldorf.de

**Keywords:** oral cancer, weight loss, quality of life, nutrition, diet, swallow

## Abstract

Diet is important for both quality of life (QoL) and survival of patients with oral cancer. Their intake of food is impeded by functional restrictions in chewing and swallowing. In the DÖSAK REHAB STUDY 1652 patients from 38 hospitals within the German-language area of Germany; Austria and Switzerland were examined with regard to functional and psychological variables having an impact on diet. Chewing and swallowing are correlated with mobility of the tongue and the mandible as well as opening of the mouth. Thirty five percent of the patients lost weight; 41% maintained their weight and 24% gained weight. The QoL of patients who were able to maintain their weight and of those who gained weight was significantly better than that of patients who lost weight. A normal diet was important for maintaining weight. Mashed food; liquid food and loss of appetite were closely associated with loss of weight; although it was possible for nutritional counseling and dietary support to be implemented particularly favorably in this respect. Due to problems with eating patients’ strength deteriorated; thus restricting activity. Radiotherapy had a negative impact on diet and weight. It influenced sense of taste; dryness of the mouth; swelling and discomfort when ingesting food. Pain and scars in the region of the operation also cause patients to dislike hard; spicy and sour food. Support from a nutritional counselor in implementing a calorie-rich diet remedied this and such support needs to be integrated into patient management. The fact that a poor nutritional status is of such great importance is well-known; but what is often lacking is the systematic implementation of continued professional nutritional counseling over a long period of time; weight control and psycho-social support of the operated patients; particularly those who also have had radiotherapy.

## 1. Introduction

Insufficient calorie intake leads to malnutrition and loss of weight in patients with oral cancer [1,2,3,4,5,6,7]. As a consequence of this, patients have more difficulty in coping with the negative impact of disease and treatment [8]. The chances of survival are diminished [9,10]. The reasons for malnutrition are to be found in functional impairments. These concern facial muscles and defects in the chewing apparatus [11,12,13,14]. However, malnutrition can also arise due to negative personality variables, for example attitude to coping with disease and negative future expectations [15,16,17,18]. The importance of a change in weight is not underestimated but remedial measures are not sufficiently implemented in patients’ management. Only recently has it been pointed out that it is important to document loss of weight over a long period of time and that a single theoretical nutritional counseling session is not sufficient [6,19,20]. Furthermore, the nutritional counselor must work together with the patient and family members until the practical implementation is embedded in the patient’s everyday routine [21]. Functional impairment of facial muscles, tongue and temporomandibular joint make it more difficult for the patient to chew and swallow [18]. These problems can be alleviated by special physiotherapeutic measures, for which measuring methods and also therapy instructions are available [22,23,24]. However, they need to be systematically integrated into the therapy. This requires interdisciplinary cooperation going beyond surgical, radiotherapeutical and rehabilitative reconstruction, which necessitates a great deal of work in comprehensive patients’ management. Neither are patients sufficiently and continuously informed about the consequences of radiotherapy, for example dryness of the mouth and how to reduce it, although these are important with regard to weight [21,25]. Not only does the diet need to be enriched with more calories but also adjusted to the patient’s needs [20,26]. A loss of teeth and problems with dental prostheses also play a part, the relevant facts need to be checked over a long period of time [14,27,28]. Overall, patients need support when they are supposed to change their behavior, whether it be in their diet or in methods of preparing food and its ingestion.

Loss of weight and malnutrition have a negative impact on quality of life and lead to patients having a gloomy view of their future [28,29,30,31]. They feel weak and tend to restrict their activities and avoid appearing in public [5,6,32]. Psychological support frequently fails because of resistance from patients so that care by specially trained medical staff is often the chosen means [33,34,35]. But coping with the disease is not the only thing to be dealt with; attitude towards diet, reliability in keeping the regular check-up appointments and checking on chewing and swallowing functions all have to be discussed with the patient. The patient’s family or caregivers need to be included when dealing with the patient’s attitude to their disease and the coping strategies which have thus become necessary [36].

## 2. Methods and Materials

Thirty-eight clinics in Germany, Austria and Switzerland participated in the multi-center retrospective DÖSAK REHAB (REHABILITATION) study of tumors in the maxillofacial region. An ethics approval was performed in every participating clinic successfully. The Bochum patient questionnaire on rehabilitation containing 147 questions in nine chapters (personal data, course of disease prior to treatment, during treatment and post-treatment, coping with disease, life circumstances and lifestyle) was used. The doctor’s questionnaire attached to each patient questionnaire included questions about tumor size, localization, neck dissection and reconstruction. Tumor size was determined according to the UICC classification of malignant tumors (1987): T1 ≤ 2 cm, T2 > 2 to 4 cm, T3 > 4 cm, T4 infiltrating neighboring structures. 1761 questionnaires were returned anonymously. The data was analyzed with the SPSS program 21.0 including descriptive statistics, correlations, chi-square test and ANOVA calculations and with a step-by-step regression analysis. The questionnaires were checked for systematic and non-systematic errors to avoid bias. A five-point Likert scale was used to measure 19 impairments (Table 1) which are important from the experience of surgeons in the Department of Maxillofacial Surgery and further symptoms that arose throughout the disease and therapy (no impairment = 0, slight impairment = 1, moderate impairment = 2, severe impairment = 3, very severe impairment = 4). Quality of life was measured using a 100-point scale (from 0 = completely dissatisfied to 100 = completely satisfied).

**Table 1:** Nineteen Impairments of patients.

Impairment of …
Eating/swallowing
General condition
Appearance (cosmesis)
Understanding of patient’s speech to strangers
Mobility of the mandible
Mouth opening
Pain
Appetite
Sense of taste
Understanding of patient’s speech to familiar people
Mobility of the tongue
Breathing
Mobility of the neck
Dryness of the mouth
Gastric disorders
Mobility of the shoulder
Swelling
Sense of smell
Halitosis

Quality of life was measured using a 100-point scale and the patients classified in three groups (very dissatisfied, satisfied, very satisfied). High standard residues (SR) indicate the closeness of the connections between two variables. The psychological variables were measured using German versions of the following scales in their short forms: depressiveness with the Depression Scaleby *vs.* Zerssen D (Depression Scale 1976; published by Hogrefe) [37], fear with STAI by Laux (State-Trait Anxiety Inventory 1972; published by Hogrefe) [38], coping with the disease with the Freiburg Questionnaire on Coping with Disease by Muthny (Freiburg Questionnaire of Coping with Disease 1996; published by Beltz/Hogrefe) [39]. Higher figures indicate a greater mental strain. The 1652 patients from the total random sample were divided into three groups: those who had lost weight, gained weight or maintained the same weight. Besides this, the groups of patients who had lost or gained weight were sub-divided into those who had lost or gained up to 10 kilograms in weight and those who had lost or gained more than 10 kg.

## 3. Results

Out of the total of 1652 patients 1526 are available concerning change in weight. Seventy five percent were men. Fifty-three Patients (3%) were 40 years and younger, 829 patients (52%) were 41 to 60 years, 594 patients (37%) were 61 to 75 and 114 (7%) patients were 76 years and older. More than one-third of the patients lost weight (Table 2). The largest group consisted of patients whose weight remained the same and a quarter of the patients gained weight. With regard to diet the differences are highly significant: within the normal diet group, 46% maintained the same weight, which is significantly the highest part. Within the liquid food group, most patients (61%) lost weight; within the mashed food group, the majority also (51%) lost weight (Table 3). Most of the patients who lost weight have to eat liquid or mashed food. Nose-stomach tube group consist of only 15 patients. This may explain the lack of significant findings. The PEG group comprises only 46 patients, which probably explains the lower statistical difference.

**Table 2:** Frequency of changs in weight.

Frequency	*N*	%
Lost weight (a)	531	35
Same weight	624	41
Gained weight (b)	371	24
Total	1526	100
Missing	126	
Total	1652	

**Table 3:** Change in weight and type of diet at the time of the study at least six months after the operation.

Present Diet	Change in Weight			Significance
	Lost Weight	Same Weight	Gained Weight	
Normal food	27%	46%	27%	*p* < 0.001
Liquid food	61%	25%	14%	*p* < 0.001
Mashed food	51%	29%	20%	*p* < 0.001
Nose-stomach tube				not significant
PEG	61%	26%	13%	*p* < 0.001

### 3.1. Classification of Change in Weight in Groups

The “lost weight” group (a, Table 2) was sub-divided into patients who had lost up to 10 kg and those who had lost 10 kg or more. The patients with a loss of weight of up to 10 kg differ from the group of patients with a greater loss of weight. The patients with a gain in weight do not differ regardless of the number of kilograms. The patients who had lost up to 10 kg were more frequently able to eat a normal diet compared with patients who had lost more than 10 kg (*p* < 0.001). Patients who lost more than 10 kg more frequently had to eat mashed food (*p* < 0.001).

The small group of 15 patients who still had a nose-stomach tube at least six months after the operation are equally distributed among the groups “gained weight”, “same weight” and “lost weight” (n.s. *p* < 0.506), which is to be seen in relation to the fact that calorie intake was determined externally. The number of PEG patients six months after the operation amounted to 3% of the total random sample. Astonishingly, these patients much more frequently belong to the group of those who lost weight (*p* < 0.001). In these cases as well, calorie intake was regulated externally.

Out of the 19 impairments the following factors were important for the amount of weight lost according to Pearson (Pearson chi-square): eating/swallowing (*p* < 0.001), mobility of the tongue (*p* < 0.001), mobility of the mandible (*p* < 0.003), mouth opening (*p* < 0.008) and dryness of the mouth (*p* < 0.054), strength (*p* < 0.001), appearance (*p* < 0.001) and speech (*p* < 0.003). But the other impairments were significant as well in their correlation with the loss of weight, even if to a lesser extent.

The following somatic variables were of importance in the group with weight loss of more than 10 kg: more lost teeth correlated with greater loss of weight (*p* < 0.001) and lower satisfaction with dental prostheses (*p* < 0.007) was also more frequently associated with greater weight loss. Small tumors T1 ≤ 2 cm were greater in number in the group of patients who had lost under 10 kg (*p* < 0.044) and correspondingly also in patients who had only undergone an operation (*p* < 0.001). Patients who lost more weight differed in their behavior regarding public appearances, on the one hand because of their speech impairment (*p* < 0.019) and on the other because of their appearance (*p* < 0.023).

### 3.2. Size of Tumor, Form of Treatment and Change in Weight

The size of the tumor determines the change in weight. Forty-eight percent of the patients with small tumors T1 ≤ 2 cm maintain their weight. Forty-five percent of the patients with tumour size T4 (infiltrating neighboring structures) lose weight and only 13% can maintain their weight. As the size of the tumor is linked to the form of treatment, the differences are also highly significant (*p* < 0.001). The group of patients who only underwent an operation did best with 47% maintaining their weight. On the other hand, the three groups (1) operation and radiotherapy; (2) operation and chemotherapy; and (3) operation, chemotherapy and radiation come off worse. Only a third of the patients in these groups can maintain their weight, more than 40% lose weight and only a quarter show a gain in weight.

Radiotherapy was the reason that patients much more frequently ate mashed or liquid food (*p* < 0.001 Pearson chi-square). Patients who underwent radiotherapy suffered a loss of weight significantly more frequently compared with other forms of treatment. Patients who only underwent an operation ate a normal diet considerably more frequently (*p* < 0.001 Pearson chi-square). Radiotherapy had a negative impact on dryness of the mouth (*p* < 0.001), sense of taste (*p* < 0.001) and swelling (*p* < 0.009).

### 3.3. Loss of Teeth and Change in Weight

The number of teeth lost during therapy has a significant negative impact on weight (*p* < 0.001). The number of teeth lost during treatment is also important for diet. In particular, patients who have lost more than 10 teeth during treatment have to eat mashed and mainly liquid food (*p* < 0.001). Patients’ satisfaction with their dental prostheses also plays a crucial part for their weight (*p* < 0.001). The worse the patients cope with their prostheses, the more weight they lose. In the group of patients who are satisfied with their dental prostheses 50% maintained their weight. In contrast, only 23% of the patients with loss of weight manage well with their prostheses.

### 3.4. Chewing/Swallowing, Normal Diet and Other Patient Impairments 6 Months after Operation

The most important factor for patients being able to eat a normal diet is no impairment in chewing and swallowing (Table 4). A negative correlation means that the more impairments patients have in chewing/swallowing the more unlikely it is that they can eat a normal diet. Chewing/swallowing is positively correlated with the other impairments, the most important of which are listed in Table 4. When patients are compelled to eat mashed or liquid food, there is a positive correlation with impairments in chewing/swallowing.

**Table 4:** Correlations between eating/swallowing and other impairments at least six months after the operation (coefficient of correlation: *i.e.*, −0.349 ** highly negative significant correlated to eating/swallowing; 0.549 ** highly positive correlation between eating/swallowing and Mobility of the tongue).

Normal Diet at Present	−0.349 **
Mobility of the tongue	0.549 **
Understanding of patient’s speech to strangers	0.544 **
Understanding of patient’s speech to familiar people	0.493 **
Mobility of the lower mandible	0.487 **
Mouth opening	0.480 **
Strength	0.461 **
Sense of taste	0.443 **

### 3.5. Discomfort with Specific Foods

Discomfort with specific foods is experienced by 25% of patients. No specific substances were mentioned but rather the consistency of the food. The greater the impairment in eating and swallowing, the more pronounced is the discomfort with some kinds of food (*p* < 0.001). Xerostomia increases aversion to several kinds of food (*p* < 0.001). As a connection already existed between stomach disorders and dislike of certain foods before treatment of oral cancer (*p* < 0.003), the significant connection between the two variables six months after the operation (*p* < 0.001) may be an indication of co-morbidity or food preferences. Pain in the region of the oral cavity and a feeling of numbness in the tongue as well as formation of scars in the region of the face and neck also cause discomfort with certain kinds of food (*p* < 0.001). If the foods mentioned by the patients are classified in groups, in particular hard, hot, sour and spicy foodstuffs are disliked.

### 3.6. Psychological Variables, Quality of Life, Future Prospects and Pain

**Figure 1:**
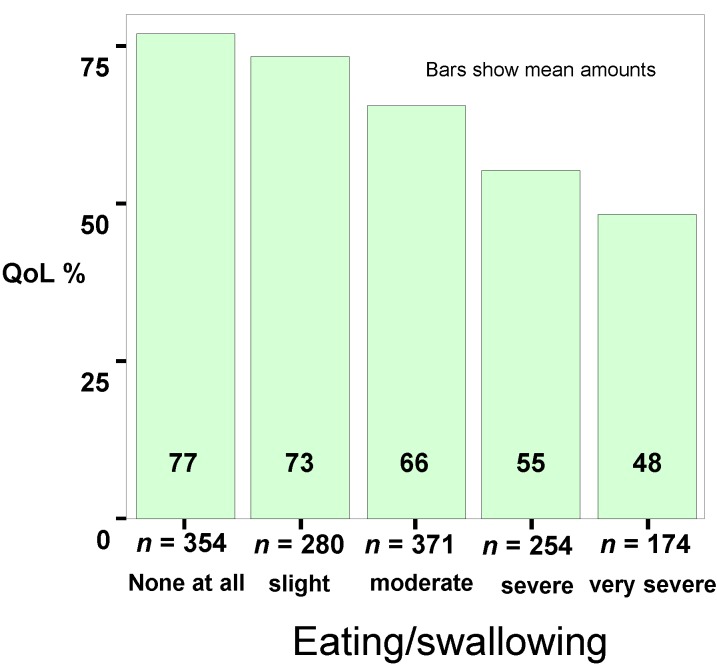
Connection between quality of life (QoL %) and impairment in eating and swallowing.

**Figure 2:**
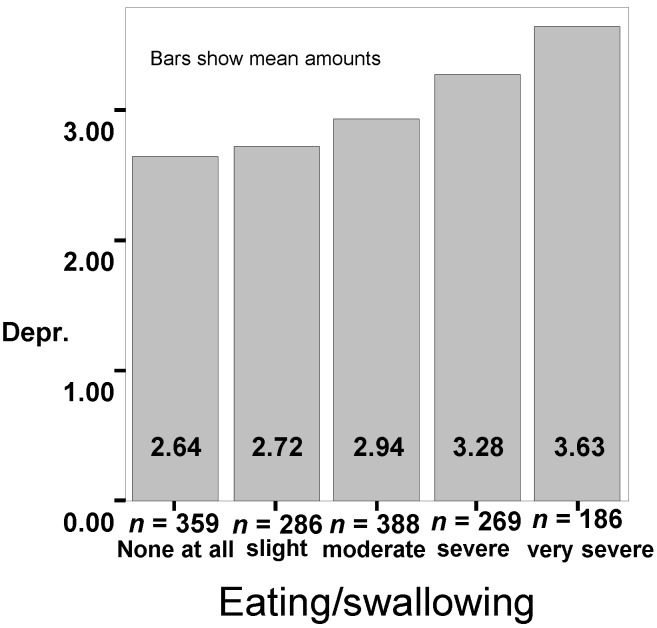
Connection between depressiveness (Depr.) and impairment in eating and swallowing.

Depressiveness and changes in weight are closely associated (*p* < 0.001). Forty six percent of the patients were able to maintain their weight after becoming ill if they did not show any depressive symptoms. Patients who showed signs of depression lost more weight. Anxiety produced the same results (*p* < 0.005). A depressive coping with their disease (Freiburg Questionnaire on Coping with Disease) was more frequent in patients with a loss of more than 10 kg in weight (*p* < 0.03). Patients with loss of weight indicated a low quality of life while patients who were able to maintain their weight were often very satisfied with their quality of life (*p* < 0.001). Patients who have lost weight are not very hopeful about the future (Standard residues (SR 3.7), *p* < 0.001) whereas patients who have gained weight are more hopeful (SR 2.1). Impairments in chewing/swallowing are significantly linked to quality of life (*p* < 0.001; chi-square test Pearson; see Figure 1), depressiveness (*p* < 0.001 chi-square test Pearson; see Figure 2) and anxiety (*p* < 0.001; chi-square test Pearson).

Those who had lost weight much more frequently expressed a wish for further cosmetic surgery (SR 3.4, *p* < 0.001). In this connection, the extent of the scar formation in the area of the operation is also to be considered: with loss of weight pronounced scarring leads to a wish for more cosmetic surgery (*p* < 0.001). Patients who have lost weight also complain more about pain in the area of the operation (oral cavity SR 3.3; temporomandibular joint SR 3.5; face SR 3.1, neck SR 2.9; shoulder SR 2.7 all *p* < 0.001). Patients with severe loss of weight are more likely to avoid appearing in public today because of impairments in eating, speech and appearance (*p* < 0.001). Under consideration of the 19 impairments and in addition of somatic variables, our regression analysis revealed that the relations to changes in weight can be explained in first place by appetite, in second place by liquid food and in third place by mashed food.

## 4. Discussion

Malnutrition and loss of weight are of greatest importance for the present and future of patients with oral cancer after an operation, radiotherapy and chemotherapy. This fact is described in older literature but only recently have authors been calling for the implementation of appropriate support for patients in therapy [4,8,11,14,16,19,29,30,34,40,41,42,43,44,45,46,47]. Documentation and checking of weight loss need to be improved so as to reduce serious consequences for patients. In this respect not only the doctor’s point of view is important but also the patient’s opinion [2,3,6,10,17,21,36,42,48,49,50,51,52,53].

This study presents 1526 usable patient reports on their weight development. The grouping revealed that a large number of patients did not experience any change in weight. Particular attention should be paid to patients who lost weight—more than a third in this study. A quarter of the patients even gained weight. The number of kilograms was considerably higher for loss of weight than for gain in weight. Because of the importance of the problem the group was subdivided into those who lost more and those who lost less than 10 kilograms. This procedure has not yet been described in the literature. Patients who lost more than 10 kg were significantly less often able to eat normal food and in general had more problems: they were dependent on mashed or liquid food, which can be objectively verified with videofluoroscopy [23]. Patients who had a PEG or nose-stomach tube rarely belonged to the group with severe weight loss as their calorie intake was regulated externally but none of these patients should lose weight. The medical staff is responsible for the right caloric intake. It should be easy to correct the situation of PEG patients losing weight, even though it was not so much. PEG has also been studied in other publications on small numbers of patients [50,51,52,54]. Conclusions from the results are still uncertain both regarding the start of the PEG treatment prior to or following the operation and also concerning the result [32]. Good training for all patients and caregivers concerning diet and weight checking is considered necessary both at the start and during the further course of therapy so as to organize rehabilitation in an optimal way [22]. A single session of nutritional counseling is not sufficient. Practical guidance may need to be given [36]. Poor nutritional status and thus loss of weight is rarely to be found with tumors of size T1 in our patients and more often in patients with greater tumors. This corresponds to the findings of other studies [1,19,29,34,49,55].

A severe loss of weight is caused among other things by loss of teeth and dissatisfaction with the fit of the dental prostheses [44,56,57]. The worse patients manage with their prostheses, the more weight they lose. Patients with severe loss of teeth after treatment of oral cancer also need greater care because of psychological problems than patients who have lost teeth for other reasons [17,27]. Diet also depends on the number of teeth lost. In particular, patients who have lost more than 10 teeth have to eat mashed and liquid food. Nutritional errors may occur as patients and their caregivers have no experience with this, although it is easy to use high-calorie additives. However, it is overall of great important to preserve teeth. Furthermore, the stability of the dental prostheses needs to be improved to safeguard the facial status of the patient concerned [58].

Chewing and swallowing are of crucial importance for the nutritional status and weight of the patient particularly in the case of radiotherapy [11,14,15,24,28,47,59,60,61]. As chewing and swallowing are correlated with other impairments, they are also of importance for nutritional status and weight and must be given attention to. When diet consists of normal food at the time of study, a positive influence on weight is to be expected [17]. A step-by-step regression analysis mentioned loss of appetite as the most important factor in first place, liquid food in second place and mashed food in third place to explain the relations to the changes in weight [22,26,30,60,62]. Nutritional counseling can be used to good effect here by providing calorie-enriched mashed or liquid food [20].

The correlation calculation revealed close connections between chewing and swallowing and mobility of the tongue, mobility of the mandible and mouth opening. This implies that physiotherapy of chewing, swallowing and transport of food can improve the patient’s situation [12,15,17,18,40]. Further links exist to speech impediments, which in turn impact the patient’s quality of life [13,63,64,65,66]. Also the patient’s strength and activity are influenced by restrictions in chewing and swallowing [21,67,68]. Poor nutritional status can result, among other things, from an impairment in the sense of taste [22,34] and from discomfort with certain kinds of food, in which dryness of the mouth plays an important part, particularly with the intake of dry food in a normal diet [61,68,69,70,71].

In this respect scar formation in the region of the operation has a negative impact as does pain in the region of the oral cavity. In summary, in particular hard, hot, sour and spicy foods are disliked while soft and cool food is preferred [20]. Patients with problems in chewing and swallowing have a reduced quality of life. This finding is frequently reported in the literature and agrees with our results [16,29,40,41,44,63,72,73,74,75]. Patients who were able to maintain their weight showed themselves to be very satisfied with their QoL [76]. Depressive symptoms were important in connection with the loss of QoL as well as anxiety and the style of coping with the disease [31,34,77,78]. Patients who lost weight are not hopeful about the future unlike those who gained weight. Psychological support might be helpful. Those who have lost weight more frequently express a wish for further cosmetic surgery. Also in this connection, scar formation and pain in the area of the operation are associated with QoL [17,40,79]. Patients with severe weight loss more frequently avoid appearing in public because of impairments in eating and appearance [68].

Psychosocial variables are occasionally more important for the patient’s assessment of their QoL than medical facts [35,58,78,80].

## 5. Conclusions

Malnutrition and loss of weight in patients with oral cancer are a result of insufficient intake of calories. The reasons are of both functional and mental nature. The consequences of weight loss are a poor style of coping with disease and only a slight prospect of survival. Sufficient attention is not always paid to the patient’s weight situation prior to and following treatment. Weight needs to be checked and documented regularly and over long periods of time and measures taken in cases of weight loss. Malnutrition sometimes results from the patient’s ignorance of nutritional facts. This needs to be rectified through information and nutritional counseling. Nutritional counseling should not consist of a single session but of guidance of an individualized nature over a long period of time. It can be both theoretical and practical. It must be ensured that the family members are included, particularly when they are in charge of providing the patient’s meals. The food not only needs to be calorie enriched but its consistency adapted to the patient’s needs as well. Appropriate programs should enable patients to cope better with their functional impairments, for example in chewing and swallowing. The fit and stability of dental prostheses and loss of teeth also require more consideration. Psychological support is advisable to cure anxiety, depressiveness avoiding other people and lack of appetite. If need be, the indication for performing a PEG should be eased, particularly as it is connected with lower morbidity.
